# Alkaline phosphatase affects renal function in never-treated hypertensive patients: effect modification by age

**DOI:** 10.1038/s41598-020-66911-z

**Published:** 2020-06-17

**Authors:** Angela Sciacqua, Giovanni Tripepi, Maria Perticone, Velia Cassano, Teresa V. Fiorentino, Gerardo N. Pititto, Raffaele Maio, Sofia Miceli, Francesco Andreozzi, Giorgio Sesti, Francesco Perticone

**Affiliations:** 1 Department of Medical and Surgical Sciences, University Magna Græcia of Catanzaro, Catanzaro, Italy; 2 CNR-IFC, Istituto di Fisiologia Clinica, Clinical Epidemiology and Physiopathology of Renal Diseases and Hypertension, Reggio Calabria, Italy; 3 Department of Experimental and Clinical Medicine, University Magna Græcia of Catanzaro, Catanzaro, Italy; 4 ASST Sette Laghi, Internal Medicine Unit, University of Insubria, Varese, Italy; 5 Department of Clinical and Molecular Medicine, University of Rome-Sapienza, Rome, Italy

**Keywords:** Biomarkers, Medical research

## Abstract

Several studies in patients with chronic kidney disease or normal renal function have shown that high levels of tissue non-specific alkaline phosphatase (ALP) are associated with an increased risk of all cause and cardiovascular (CV) mortality. Considering the independent prognostic role of renal function, we investigated the possible association between ALP levels and estimated glomerular filtration rate (e-GFR) in a large cohort of hypertensive subjects. We enrolled 2157 never-treated uncomplicated hypertensive patients with ALP levels within normal range. In the whole population, e-GFR was strongly related to ALP (r = −0.43, P < 0.0001) with similar magnitude in females and in males, resulting ALP the second independent predictor of renal function. In a multiple linear regression model, both on crude (P < 0.001) and adjusted (P = 0.01) analyses age significantly modified the effect of a fixed increase in ALP (20 UI/L) on renal function so that the reduction in e-GFR associated to a 20 UI/L increase in ALP was of lower magnitude in younger patients and progressively of higher extent from 20 years of age onwards. In conclusion, present data indicate a significant relationship between ALP levels and e-GFR in uncomplicated hypertensive patients that is modulated by age and that persisted after adjusting for several confounders.

## Introduction

Tissue non-specific alkaline phosphatase (ALP), routinely measured in clinical practice for the diagnosis and monitoring of bone and hepatobiliary diseases, is a widely expressed hydrolase enzyme in human tissues, particularly in the liver, bone and kidney. The main action of ALP is to catalyse the hydrolysis and therefore the inactivation of inorganic pyrophosphate (PPi), a potent endogenous inhibitor of calcification^1^

Several evidences suggest a possible role of ALP as emerging marker of cardiovascular (CV) risk. In particular, numerous studies in patients with chronic kidney disease (CKD) have shown that high levels of ALP are associated with an increased risk of CV and all-cause mortality^2–4^. In addition, high values of ALP are associated with increased risk of kidney disease progression in a heterogeneous population of patients with CKD stages 3-4^5^. Moreover, ALP has been demonstrated to be an independent predictor of death and CV events in both the general population and patients with coronary artery disease (CAD) with preserved renal function^6,7^.

Several possible mechanisms have been hypothesized to explain the possible role of ALP in CV risk, including subclinical inflammation^8,9^, vascular calcification^10,11^, and endothelial dysfunction^12,13^. Recently, a strong association between serum ALP levels and reduced endothelium-dependent vasodilation, an independent predictor of CV events, was demonstrated in a large cohort of hypertensive patients^12^.

It is well known the strong link between renal function and CV outcome across all CKD stages. Indeed, several studies have shown that both advanced and subclinical renal damage are independent predictors of CV events and hospitalization in different settings of patients^14,15^. Of interest, experimental evidence suggests that ALP may directly affect renal function by increasing adenosine production and enhancing reno-vascular responses to norepinephrine in the kidney^16^. Moreover, subclinical inflammation and endothelial dysfunction, also promoted by ALP, may furtherly favour renal damage. However, whether serum ALP levels are associated with renal function in hypertensive patients without kidney and CV complications as well as the potential effect modification of aging on this relationship have not been settled. In the present study, this hypothesis was analyzed in a large cohort of never-treated hypertensive subjects participating in the CATAnzaro MEtabolic RIsk factors (CATAMERI) Study.

## Results

### Study population

Main demographic, clinical and biochemical data of the whole study group as well as separately by gender are reported in Table 1. There were no significant differences between males and females for ALP as well as for SBP, DBP, LDL-cholesterol and smoking. However, males were significantly older and displayed higher values of BMI, PP, fasting glucose, triglycerides, and hs-CRP than females. On the contrary, HDL-cholesterol values were significantly lower in males than in females. Furthermore, serum levels of phosphorus, calcium, and UA were significantly higher in males, so as e-GFR, that resulted normal in both groups (all eGFR values>60 ml/min/1.73 m^2^).

**Table 1:** Baseline characteristics of the study population according to gender.

	All (N = 2157)	Females (N = 1100)	Males (N = 1057)	P
Age, *yrs*	53.1 ± 13.5	50.3 ± 15.8	53.1 ± 13.5	<0.0001
Smokers, *n (%)*	425(19.7)	201(18.3)	224(21.2)	0.088
Body mass index, *Kg/m*^2^	32.1 ± 9.9	25.8 ± 4.4	32.1 ± 9.9	<0.0001
Systolic BP, *mm Hg*	137.3 ± 17.1	136.1 ± 18.5	137.3 ± 17.1	0.106
Diastolic BP, *mm Hg*	85.1 + 10.8	85.1 ± 10.8	85.1 ± 10.9	0.954
Pulse Pressure, *mm Hg*	52.1 ± 13.9	50.1 ± 14.9	52.1 ± 13.9	0.001
Fasting glucose, *mg/dL*	99.1 ± 13.4	93.9 ± 12.8	99.1 ± 12.4	<0.0001
LDL cholesterol, *mg/dL*	122.9 ± 35.7	123.9 ± 36.9	122.9 ± 35.7	0.529
HDL cholesterol, *mg/dL*	46.9 ± 13.9	53.7 ± 13.9	46.9 ± 13.9	<0.0001
Triglycerides, *mg/dL*	142.7 ± 82.4	120.4 ± 68.8	142.7 ± 82.4	<0.0001
hs-CRP, *mg/L*	3.5 ± 2.5	2.9 ± 2.2	3.5 ± 2.5	<0.0001
Phosphorus, *mg/dL*	3.5 ± 0.6	3.3 ± 0.6	3.5 ± 0.6	<0.0001
Alkaline phosphatase, *U/L*	81.8 ± 29.4	81.5 ± 34.3	81.8 ± 29.4	0.801
Calcium, *mg/dL*	9.5 ± 0.5	9.4 ± 0.5	9.5 ± 0.5	0.001
Uric acid, *mg/dl*	5.7 ± 1.3	4.5 ± 1.4	5.7 ± 1.3	<0.0001
e-GFR, *ml/min/1.73/m*^2^	95.9 ± 16.8	96.1 ± 16.9	100.8 ± 13.9	<0.0001

BP = blood high sensitivity C reactive protein; e-GFR = estimated glomerular filtration pressure; hs-CRP = rate.

### ALP and eGFR: univariate and multivariate analyses

**Figure 1:**
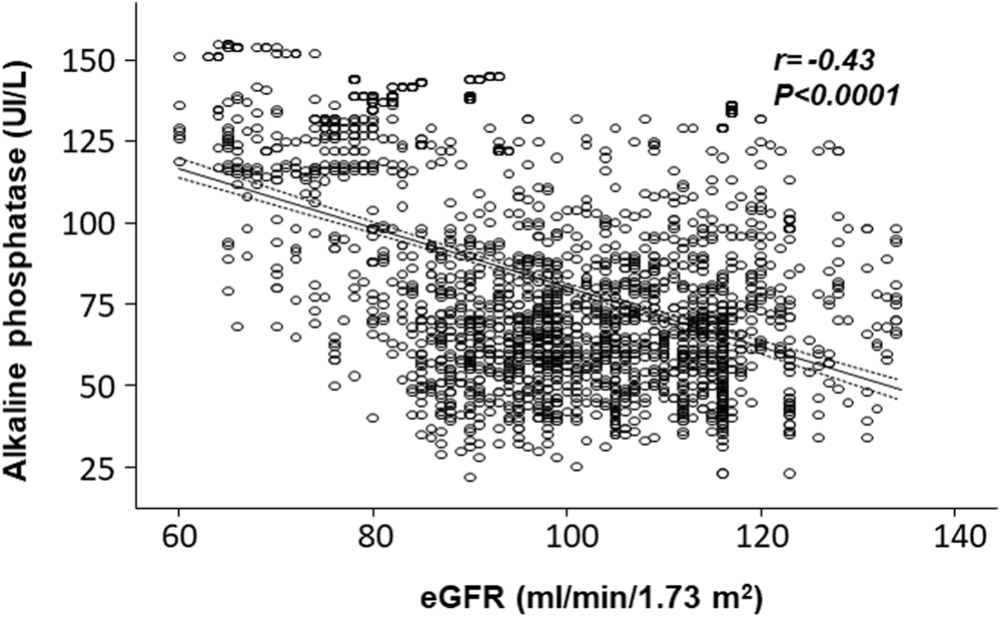
Crude relationship between estimated-glomerular filtration rate (e-GFR) and alkaline phosphatase (ALP) in the whole study population.

In the whole group, e-GFR was strongly inversely related to ALP (r = −0.43, P < 0.0001, Fig. 1), and the strength of this relationship was identical in both females (r = −0.50, P < 0.001) and males (r = −0.50, P < 0.001). Data adjustment for hs-CRP reduced the strength of the ALP - eGFR relationship (adjusted r = − 0.38, P < 0.001) suggesting that inflammation lies in the causal pathway between high ALP and low eGFR.

In a multiple linear regression model (dependent variable: e-GFR) including traditional and non-traditional risk factors, BMI (β = 0.35, P < 0.001), hs-CRP (β = −0.24, P < 0.001), male gender (β = −0.18, P < 0.001), age (β = −0.18, P < 0.001), UA (β = −0.15, P < 0.001), phosphate (β = −0.12, P < 0.001), LDL cholesterol (β = 0.07, P = 0.03), systolic BP (β = −0.04, P = 0.015) and triglycerides (β = 0.05, P = 0.005) had an independent relationship with e-GFR whereas smoking (β = 0.02, P = 0.16), and total cholesterol (β = −0.03, P = 0.36) did not (Table 2). All these variables explained 38% of the total variance in e-GFR (R^2^ = 0.38). The inclusion of ALP into this model provided a 6% increase in the explained variance in e-GFR (from 38% to 44%) and ALP (β = −0.26, P < 0.001) resulted to be the second factor, in rank order after BMI, explaining the variability in e-GFR (β = −0.26, P < 0.001).

**Table 2:** Multiple linear regression analysis on e-GFR.

	Model 1 (beta and P value)	Model 2 (beta and P value)
*R*^2^	0.38 (38%)	0.44 (44%)
Male gender	-0.18, P < 0.001	−0.18, P < 0.001
Age (years)	−0.18, P < 0.001	−0.14, P < 0.001
Smoking (0=no; 1=yes)	0.02, P = 0.16	0.03, P = 0.09
BMI (kg/m2)	0.35, P < 0.001	0.30, P < 0.001
Systolic BP (mmHg)	−0.04, P = 0.015	−0.03, P = 0.07
Total cholesterol (mg/dL)	−0.03, P = 0.36	−0.04, P = 0.17
LDL cholesterol (mg/dL)	0.07, P = 0.03	0.07, P = 0.02
Triglycerides (mg/dL)	0.05, P = 0.005	0.06, P = 0.001
Uric acid (mg/dL)	−0.15, P < 0.001	−0.12, P < 0.001
Phosphate (mg/dL)	−0.12, P < 0.001	−0.12, P < 0.001
C-Reactive protein (mg/L)	−0.24, P < 0.001	−0.20, P < 0.001
Alkaline phosphatase (UI/L)		−0.26, P < 0.001

Data are standardised regression coefficients (beta) and P value.

e-GFR = estimated glomerular filtration rate; BMI = body mass index; BP = blood pressure.

### Effect modification by age on the relationship between ALP and e-GFR: univariate and multivariate analyses

**Figure 2:**
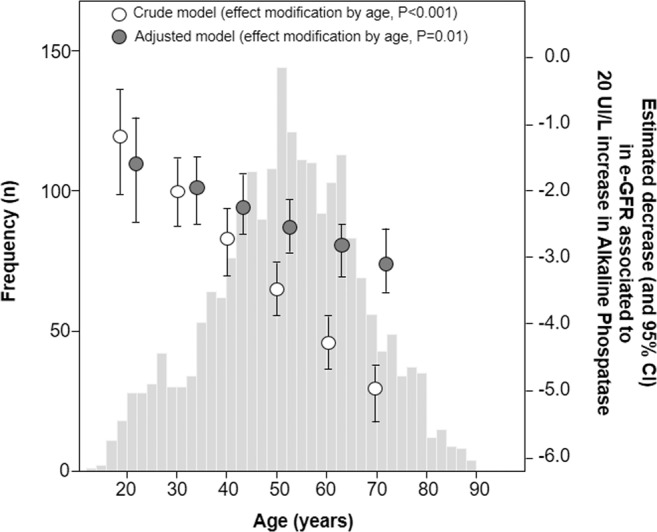
Effect modification by age on the relationship between alkaline phosphatase (ALP) and estimated-glomerular filtration rate (e-GFR). Data are expressed as estimated decrease (and 95% CI) in e-GFR associated to 20 UI/L increase in ALP at predefined values of age Equations of both crude and adjusted effect modification models: *Crude effect modification model:* e-GFR = 111.66 + 0.224*ALP_20 (20 units step)+0.041*Age (continuous variable)−0.075*interaction; where “interaction” is the product between “ALP_20” and “Age” as preliminary calculated. *Adjusted effect modification model:* eGFR= 115.68-1.070*ALP_20 - 0.032*Age - 0.029*interaction - 5.782*Gender + 1.111*Smoking +0.561*BMI - 0.025*systolic blood pressure -0.018*Total cholesterol + 0.032*LDL + 0.012*triglycerides - 1.229*uric acid -1.995*phosphate - 1.30*C reactive protein (see Methods- Statistical Analysis for more details).

On univariate analysis (P < 0.001) as well as in a multiple linear regression model (P = 0.01), including the same set of covariates listed in Table 2, age significantly modified the effect of a fixed increase in ALP (20 UI/L) on e-GFR, so that such an increase in ALP triggered a decrease in e-GFR which was closely dependent on age (Fig. 2). The analysis of the “age-ALP interaction” showed that the reduction in e-GFR associated to a 20 UI/L increase in ALP was of lower magnitude in younger patients and progressively of higher extent from 20 years of age onwards. To put this in perspective, the estimated reductions in eGFR, adjusted for risk factors listed in Table 2, associated to a 20 UI/L increase in ALP was 1.7 ml/min/1.73 m^2^ when patients were 20 years old and 3.1 ml/min/1.73 m^2^ when patients were 70 years old (Fig. 2).

## Discussion

The results of this study, conducted in a well characterized cohort of non-diabetic, newly-diagnosed, never-treated hypertensive patients without CKD or CV complications, showed an association between serum ALP levels and renal function. Indeed, an inverse correlation between ALP and e-GFR values has been demonstrated both in the whole population and in sub-groups stratified by gender. Moreover, ALP was a strong correlate of e-GFR even after adjusting for traditional and non-traditional risk factors for renal impairment.

Notably, after adjusting for several confounders, age significantly modified the effect of a fixed increase in ALP on e-GFR with a lower magnitude in younger patients, which progressively raise from 20 years of age onwards, showing an “age-ALP interaction”. This is clinically relevant because epidemiological studies have shown that even a modest impairment of renal function may be associated with CV morbidity and mortality^14,15^. For instance, in the Hoorn study a loss of 5 ml/min/m^2^ in e-GFR was associated with a 22% increase in the risk of CV death^17^. Taken together, these results may be helpful to improve the stratification of global CV risk in hypertensive patients.

Several pathophysiological mechanisms including subclinical inflammation, vascular calcification, and endothelial dysfunction may explain these findings. ALP is closely associated with CRP, a commonly used inflammatory index, and there is evidence that CRP attenuates the association between ALP and CV outcome in multivariable regression models^8,18,19^. According with this, data adjustment for CRP reduced the strength of the ALP - eGFR relationship, also in our population, suggesting an involvement in the causal pathway between high ALP and low eGFR. Thus, it is possible that low-grade inflammation, characterizing chronic clinical conditions such as essential hypertension, may play a role in the association between ALP and renal damage. In addition, vascular calcification is now recognized as an active, cell-mediated process linked to an imbalance between promoters and inhibitors of mineralization^1^. ALP is able to affect the Pi:PPi ratio, which plays an undisputed role in promoting vascular calcification. According with this, ALP activity has been associated with medial vascular calcification in experimental models^10,11^.

Moreover, the association between ALP and renal damage may be, at least in part, explained by endothelial dysfunction, a strong and independent predictor of CV events in different clinical conditions, including essential hypertension^20^. Endothelial dysfunction, characterised by a reduced nitric oxide (NO) bioavailability, may be promoted by both inflammation and vascular calcification, thus favouring renal damage. In the kidney, NO exerts multiple roles including regulation of renal haemodynamic, maintenance of medullary perfusion, mediation of pressure–natriuresis, blunting of tubule-glomerular feedback, inhibition of tubular sodium reabsorption, and modulation of renal sympathetic neural activity with the net effect of promoting natriuresis and diuresis^21,22^. An association between acetylcholine (ACh)-stimulated vasodilation and renal function decline after adjustment for traditional cardiovascular risk factors has been previously reported in a cohort of never-treated uncomplicated hypertensive patients thus pointing to an important pathophysiological mechanism linking vascular dysfunction and mild renal impairment^23^. In addition, it has been shown that endothelial vasomotor dysfunction in the brachial artery is independently associated with progression from normal to early stage of renal dysfunction within twelve months of follow-up in patients with CAD. Thus, endothelial dysfunction evaluated in the brachial artery reflects a reduction in NO activity also in the renal vasculature with a consequent decline in renal function^24^ and ALP may be an active player in this association.

Mechanisms linking ALP to endothelial dysfunction may include inhibition of tyrosine kinase activity into endothelial cells with consequent impairment of endothelial NO synthase function, promotion of high production of reactive oxygen species (ROS), and apoptosis due to increased degradation of pyrophosphate promoting atherosclerotic lesions in vascular wall^12,25–27^.

Notably, we found that age acts as modifier of the relationship between ALP and e-GFR. As matter of the fact a 20 UI/L increase in ALP was associated with 1.7 ml/min/1.73 m2 reduction of e-GFR when patients were 20 years old and 3.1 ml/min/1.73 m2 when patients were 70 years old. It is known that ageing is associated with a proinflammatory status that is characterized by high levels of pro-inflammatory markers, a condition named “inflammageing”, that represents a potential risk factor for CV and renal diseases^28,29^. In hypertensive patients, fibrosis, perivascular inflammation with increased ROS production, and arterial calcification may be favoured not only by ageing but also by ALP activity, thus promoting vascular damage in both the micro- and macro-circulation in different organs and tissues, including kidney^30,31^.

Finally, recent evidence supports the notion that ALP is capable to modulate renal function and haemodynamic parameters. ALP has been involved in the regulation of renovascular and blood pressure responses to norepinephrine by modulating adenosine production in the kidney^16^. Endogenous adenosine, through the activation of A_1_-receptors highly expressed in the pre-glomerular microcirculation, is able to modulate the renovascular responses to renal sympathetic nerve stimulation as well as to exogenous norepinephrine. In the renal vasculature, in fact, adenosine and norepinephrine synergistically activate phospholipase C, contributing to renal vasoconstriction induced by sympathetic stimulation^32,33^. On the other hand, ALP is able to catalyse the conversion from ATP to adenosine in various organs including the kidney as evidenced by studies with isolated, perfused rat kidneys showing that the use of ALP specific inhibitors can attenuate the renovascular response to both exogenous and endogenous norepinephrine^16^.

The study strengths include the large number of patients enrolled with a complete clinical and laboratory assessment. Moreover, we included only never-treated patients without liver, bone, or renal disease, diabetes or excessive alcohol intake thus excluding potential confounding conditions. In addition, measurements of ALP and other biomarkers were made in a single laboratory using fresh collected blood samples. However, there are some limitations that should be considered. First, the cross-sectional design of the study does not allow to conclude a causal relationship between ALP and renal function and to define which pathophysiological mechanism plays a main role in this association. Furthermore we can not exclude that e-GFR could act also as determinant of ALP, so as evident in the advanced stages of CKD. In addition, even if our study population shows a relatively normal renal function, another important limitation is that we did not measure fibroblast growth factor (FGF)−23 and parathyroid hormone levels. These represent two well-known biomarkers of CKD and bone metabolism, thus they could affect ALP increase.

Further prospective and interventional studies are needed to confirm the observed relationship. Additionally, the present results were obtained in hypertensive patients and further studies in different patients’ settings are necessary to validate our observations. Finally, data on isoforms of ALP and measurements of mineral balance regulating factors such as vitamin D and parathyroid hormone were not available.

In conclusion, the present data indicate, in a large cohort of never-treated hypertensive patients with relatively normal renal function, a significant relationship between ALP levels and e-GFR that is modulated by age and that persisted after adjusting for several confounders. Thus, a potential involvement of ALP in the development of renal damage may be envisioned. However, further studies are needed to confirm our findings in the general population, to determine the pathophysiological mechanism, and to appraise the cause-effect relationship.

## Methods

### Study population

In this cross-sectional study, the enrolled group includes 2157 uncomplicated hypertensive outpatients (1057 males and 1100 females; mean age, 53.1 ± 13.5 years) participating in the Catanzaro Metabolic Risk Factors Study (CATAMERIS)^34,35^. All patients were Caucasian and underwent physical examination and review of their medical history. Causes of secondary hypertension were excluded by appropriate clinical and biochemical tests according to a standard diagnostic protocol. Other exclusion criteria were as follows: history or clinical evidence of CV complications, diabetes mellitus, history of any malignant disease or liver failure, calcium-phosphorus metabolism disorders, alcohol or drug abuse, and use of drugs interfering with ALP concentrations. All subjects showed a normal renal function and none of them had ever been treated with antihypertensive drugs. They underwent anthropometrical evaluation with measurement of weight, height, and body mass index (BMI). The local Ethical Committee (Comitato Etico Azienda Ospedaliera “Mater Domini”) approved the protocol and informed written consent was obtained from all participants. All the investigations were performed in accordance with the principles of the Declaration of Helsinki.

### Blood pressure measurements

Measurements of clinic blood pressure (BP) were obtained in the left arm of the supine patients, after 5 min of quiet rest, with a validated sphygmomanometer. A minimum of three BP readings were taken on three separate occasions at least 2 weeks apart. Systolic and diastolic BP was recorded at the first appearance (phase I) and the disappearance (phase V) of Korotkoff sounds. Baseline BP values were the average of the last two of the three consecutive measurements obtained at intervals of 3 minutes. Patients with a clinic systolic BP (SBP) > 140 mmHg and/or diastolic BP (DBP) > 90 mmHg were defined as hypertensive^36^. Clinical pulse pressure (PP) was measured as the difference between SBP and DBP.

### Laboratory determinations

All laboratory measurements were carried out after at least 12 hours fasting. Serum ALP (normal range, 40–129 IU/L) and calcium (normal range, 8.5–10.3 mg/dL) levels were measured with standard assays implemented on the COBAS C501 Roche auto-analyzer. The quantitative concentration of inorganic phosphate (normal range, 2.3–4.5 mg/dL) was measured by spectrophotometric determination based on the formation of ammonium molybdophosphate with subsequent reduction to molybdenum blue, using the diagnostic assay COBAS INTEGRA Phosphate 2 (Roche diagnostics). Plasma glucose was measured by the glucose oxidase method (Beckman Glucose Analyzer II; Beckman Instruments, Milan, Italy). Triglyceride, total, low- (LDL) and high-density lipoprotein (HDL) cholesterol concentrations were measured by enzymatic methods (Roche Diagnostics GmbH, Mannheim, Germany). High-sensitivity C-reactive protein (hs-CRP) was measured by a turbidimetric immunoassay (Behring).

### Markers of renal function

Serum creatinine and uric acid (UA) were measured by an automated technique based on the measurement of Jaffe chromogen and by the URICASE/POD method (Boehringer Mannheim, Mannheim Germany) implemented in an auto-analyzer. Values of e-GFR were calculated by the CKD-Epidemiology Collaboration proposed equation that is more accurate in subjects with e-GFR > 60 ml/min/1.73 m^2,37^.

### Statistical analysis

Normally distributed variables were expressed as mean ± SD and non-normally distributed variables as median and interquartile range. Categorical data were summarised as absolute numbers and percentage. Comparisons between two groups were performed by unpaired Student’s *t*-test, Mann Whitney U test, or Chi Square Test, as appropriate. The association between two continuous variables considered simultaneously was investigated by Pearson product moment correlation coefficient (r) and P values. The relationship between the key risk factor (ALP) and e-GFR was investigated by univariate (crude) analysis as well as by a multiple linear regression model including age, male gender, smoking (0=no; 1=yes), BMI (kg/m^2^), SBP (mmHg), total cholesterol (mg/dL), LDL-cholesterol (mg/dL), triglycerides (mg/dL), UA (mg/dL), phosphate (mg/dL), and hs-CRP (mg/L). To assess at what extent the inclusion of ALP into a multiple linear model (including standard risk factors – Model 1) increases the proportion of explained variance of eGFR (dependent variable), two multiple linear models were constructed: Model 1, not including ALP and Model 2, including ALP. The explained variance in the dependent variable (e-GFR) provided by tested covariates (before and after the inclusion of ALP) was assessed by the squared of the coefficient of determination (R^2^). In multiple linear regression models data were expressed as standardised regression coefficient (beta) and P value. The effect modification^38^ by age on the ALP/e-GFR relationship was investigated by the standard approach, i.e. by simultaneously introducing ALP, age and their interaction (multiplicative) term (age x ALP) into the same linear regression model also adjusting for a series of potential confounders. The estimated decrease (and 95% CI) in e-GFR associated to a fixed increase (20 UI/L) in ALP at predefined values of age was investigated by the linear combination method. All analyses were performed by two standard statistical packages (SPSS for Windows Version 22, IBM, USA; STATA/IC 13.0 StataCorp P, TX, USA).

## Data Availability

Data are available upon reasonable request from corresponding author.
